# Supplementary material for: Biological Assay-Guided Fractionation and Mass Spectrometry-Based Metabolite Profiling of Annona muricata L. Cytotoxic Compounds against Lung Cancer A549 Cell Line
Source: Plants (Basel). 2022 Sep 12;11(18):2380. doi: 10.3390/plants11182380 (PMC9503541; doi:10.3390/plants11182380)
Supplement: Supplementary file 1 [file plants-11-02380-s001.zip › plants-1885919-supplementary.pdf]

Table S1. Masses of obtained fractions from the purification of *Annona muricata*.

**Sample Code**	**Mass of Fraction (g)**	**Mass of Fraction (mg)**
1st Purification		
GE_crude	0.0028	2.8
GE_P1_B2_F17	0.0035	3.5
GE_P1_B2_F18	0.0043	4.3
GE_P1_B2_F19	0.0008	0.8
GE_P1_B2_F20	0.0016	1.6
GE_P1_B2_F21	0.0017	1.7
A	0.0002	0.2
B	0.0001	0.1
C	0.0001	0.1
D	1E-04	0.1
E	1E-04	0.1
F	0.0047	4.7
G	0.0006	0.6
H	0.0001	0.1
GE_P2_B3_F20_F16	0.0012	1.2
GE_P1_B3_F20	0.0009	0.9
F15 and F17 fractions		
GE_P3_B3_F17_12_A	0.0016	1.6
GE_P3_B3_F17_12_B	0.0005	0.5
GE_P3_B3_F17_12_C	0.0004	0.4
GE_P3_B3_F17_12_D	0.0005	0.5
GE_P3_B3_F17_12_E	0.0007	0.7
GE_P3_B3_F17_12_F	0.0011	1.1
GE_P3_B3_F17_12_G	0.0009	0.9
GE_P3_B3_F17_12_H	0.001	1
GE_P3_B3_F17_12_I	0.0003	0.3
GE_P2_B4_F17_F12	0.0018	1.8
GE_P1_B4_F17	0.0013	1.3
F15-F16 fractions		
GE_P1_B234_F15	0.0039	3.9
GE_P2_B234_F15_F9	0.0008	0.8
GE_P2_B234_F15_F10	0.0004	0.4
GE_P2_B234_F15_F11	0.0006	0.6
GE_P2_B234_F15_F12	0.0005	0.5

GE_P2_B234_F15_F13	0.0053	5.3
GE_P2_B234_F15_F14	0.0023	2.3
GE_P2_B234_F15_F15	0.0018	1.8
GE_P2_B234_F15_F16	0.0038	3.8
GE_P2_B234_F15_F17	0.0014	1.4
GE_P2_B234_F15_F18	0.0004	0.4
GE_P2_B234_F15_F19	0.0001	0.1

Alignment ID	Relative Intensity Per Fraction	Relative Intensity in Active and Inactive Fractions
13271	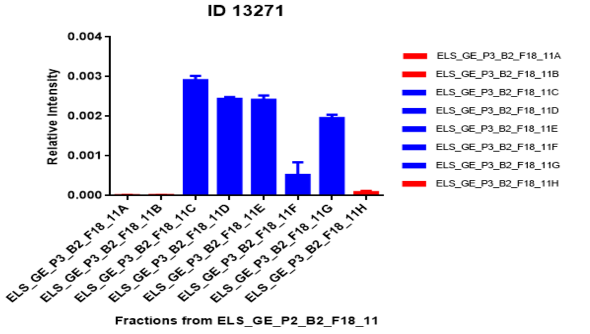	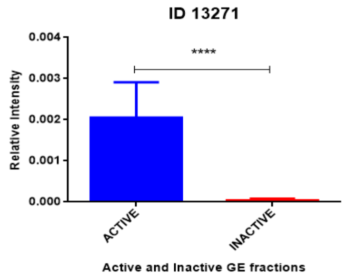
12388	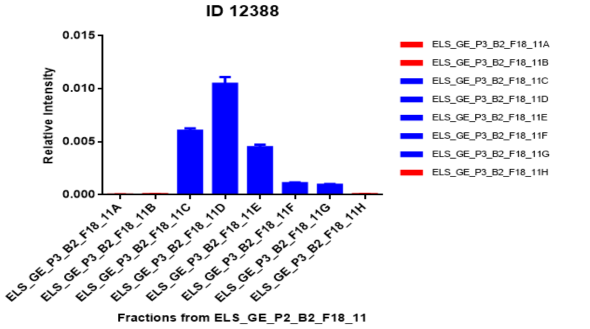	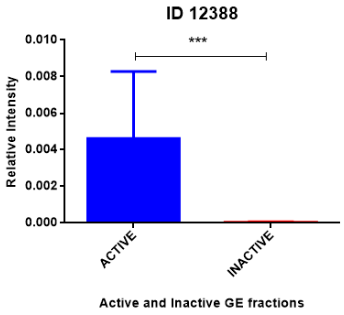
12357	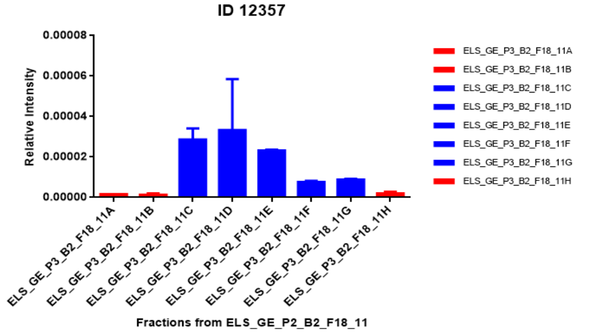	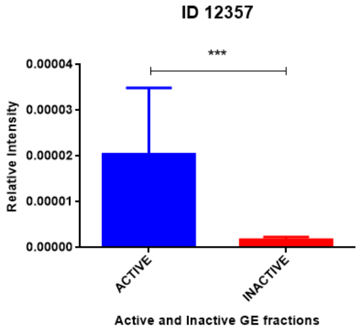
12376	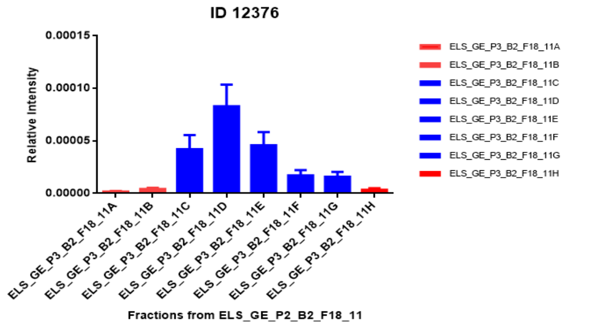	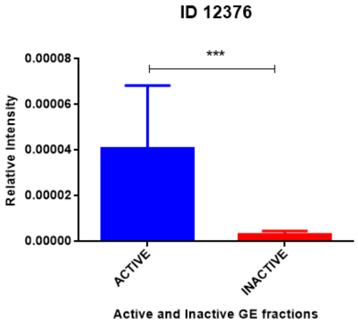

Alignment ID	Relative Intensity Per Fraction	Relative Intensity in Active and Inactive Fractions
3078	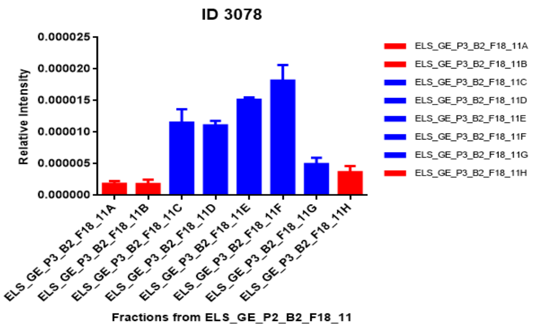	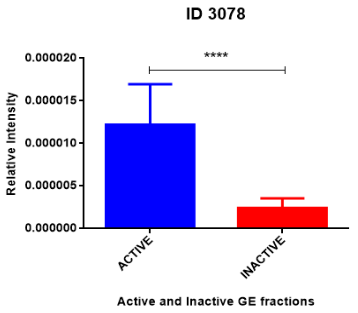
13297	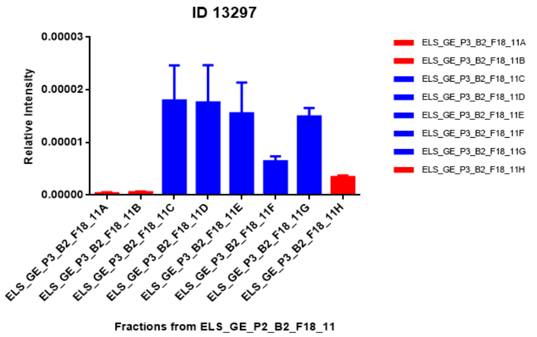	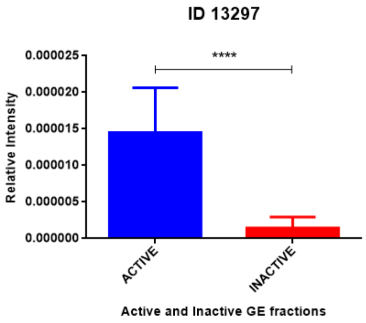
12369	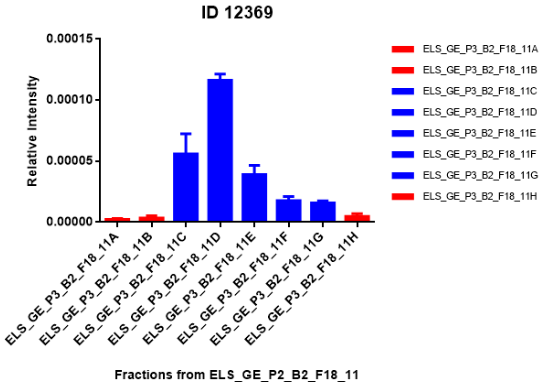	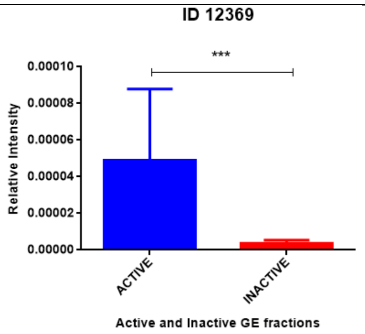

11145	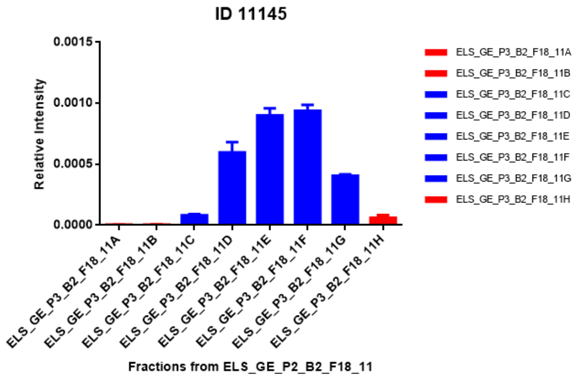	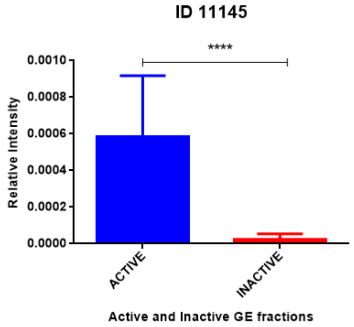
Alignment ID	Relative Intensity Per Fraction	Relative Intensity in Active and Inactive Fractions
12433	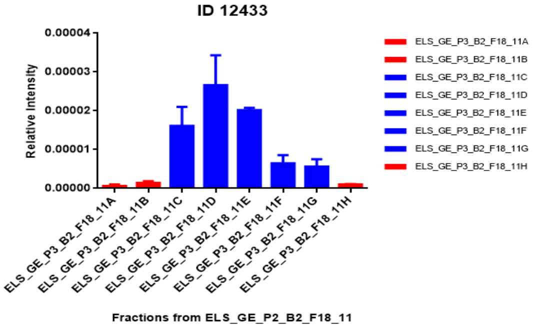	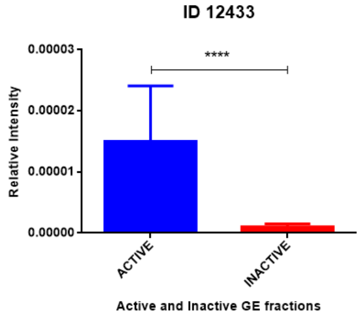
12422	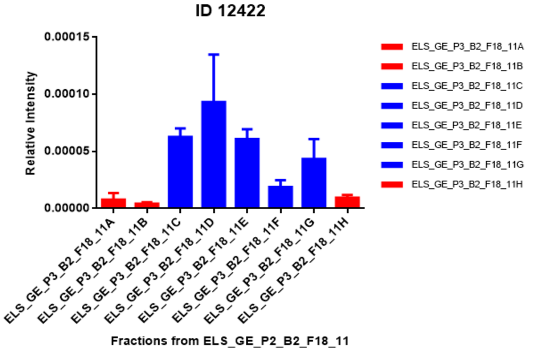	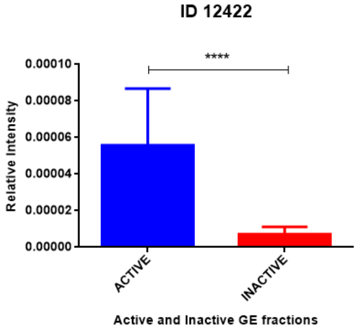
11920	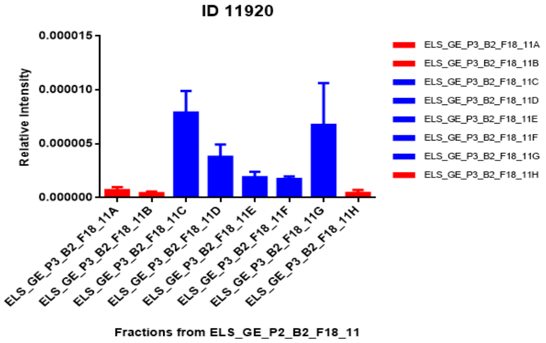	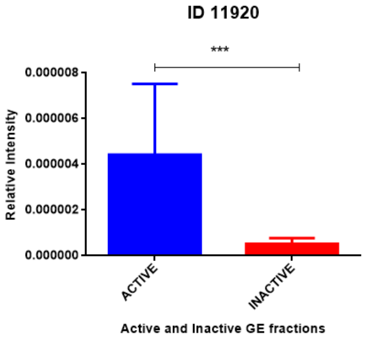

11996	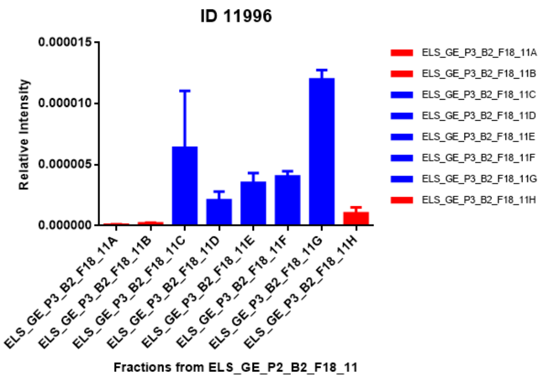	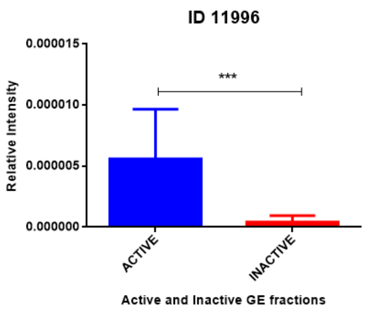
Alignment ID	Relative Intensity Per Fraction	Relative Intensity in Active and Inactive Fractions
11931	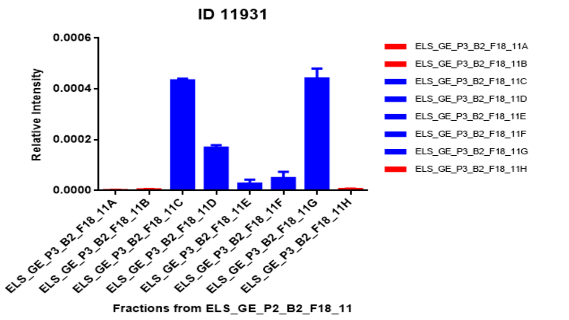	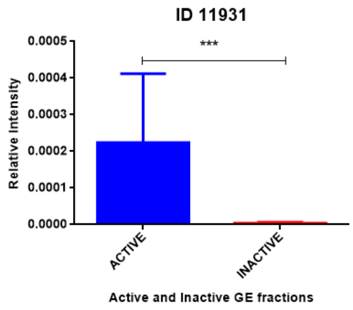
12363	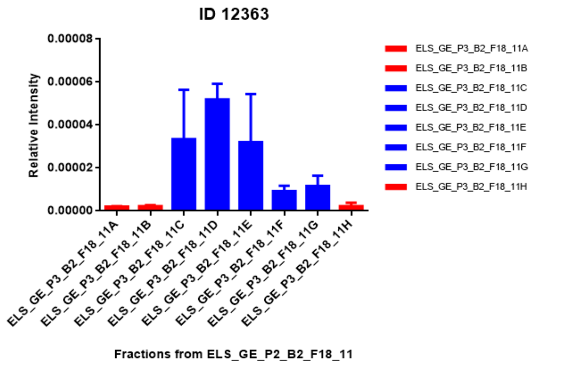	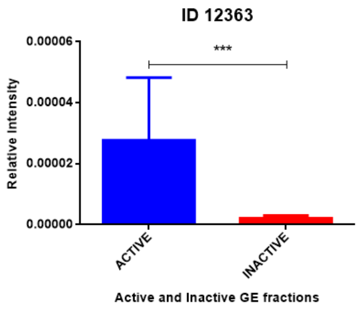
13254	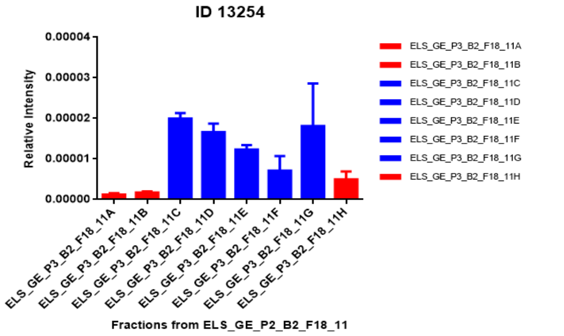	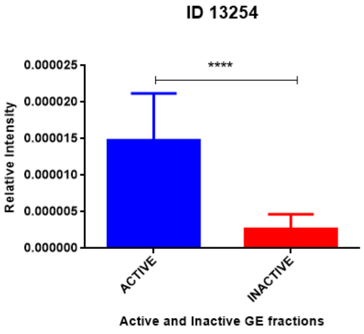

<table>
<tr><td>10417</td><td>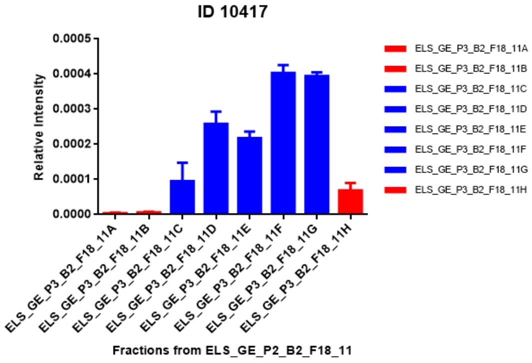
</td><td>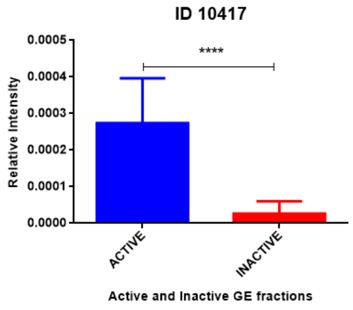
</td></tr>
<tr><td>Alignment ID</td><td>Relative Intensity Per Fraction</td><td>Relative Intensity in Active and Inactive Fractions</td></tr>
<tr><td>13144</td><td>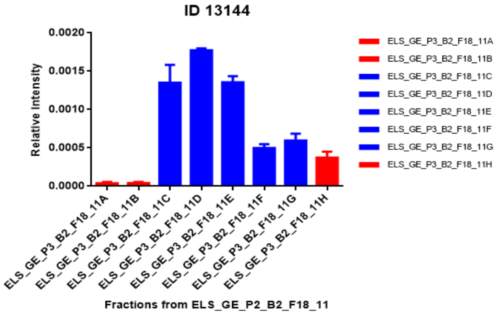
</td><td>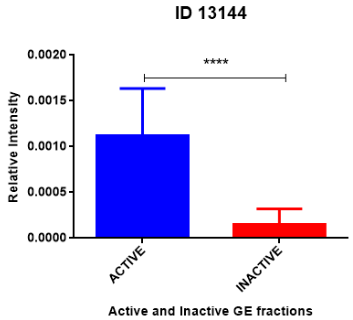
</td></tr>
<tr><td>11923</td><td>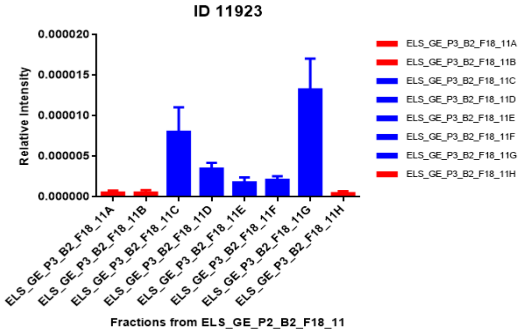
</td><td>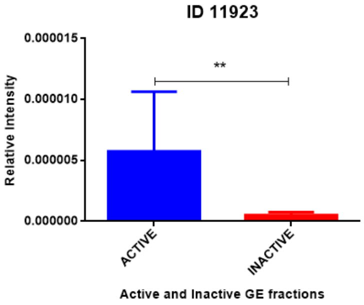
</td></tr>
<tr><td>9844</td><td>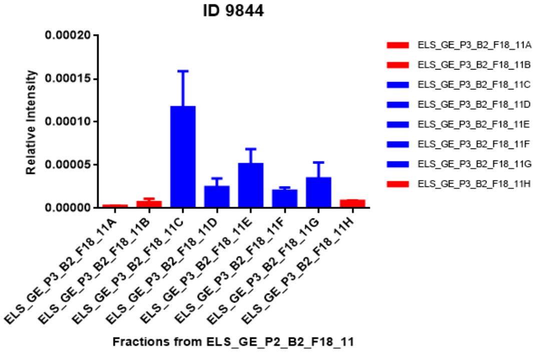
</td><td>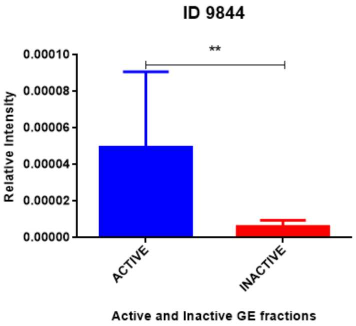
</td></tr>
</table>

11985	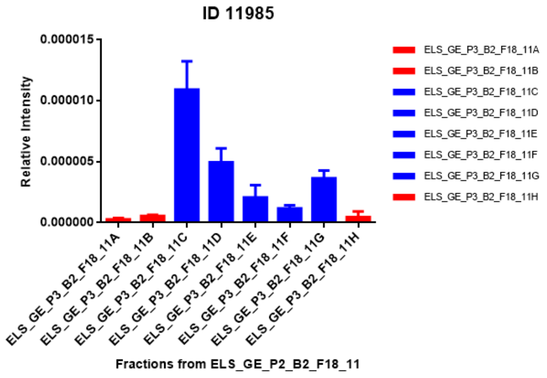	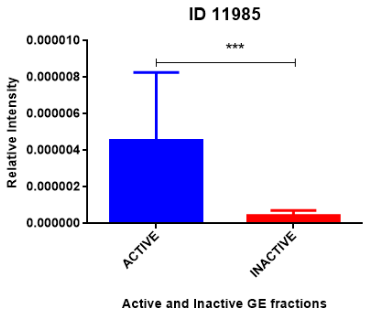

Figure S1. Putative compounds in F15-16 and their relative intensities (from MetaboAnalyst analysis)

Alignment ID	Relative Intensity Per Fraction	Relative Intensity in Active and Inactive Fractions
15623	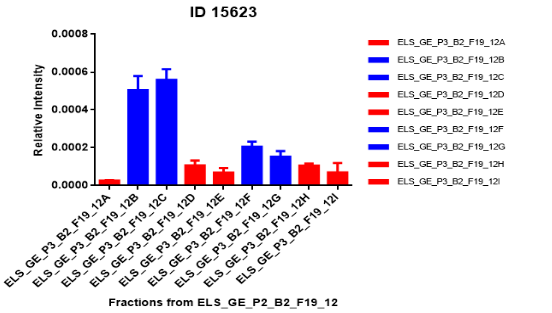	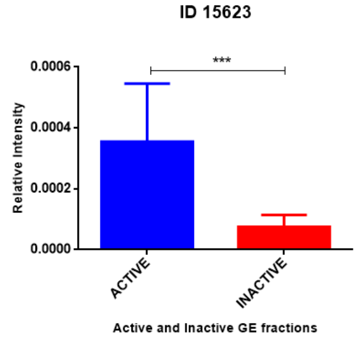
14263	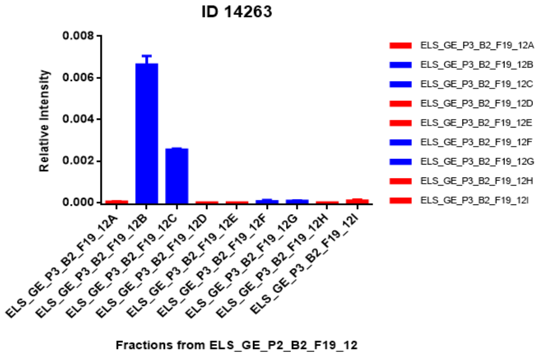	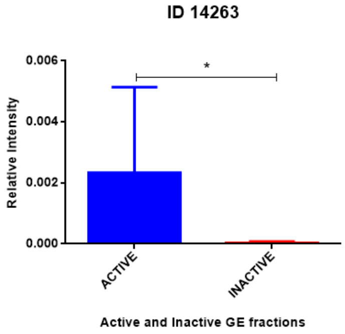
3653	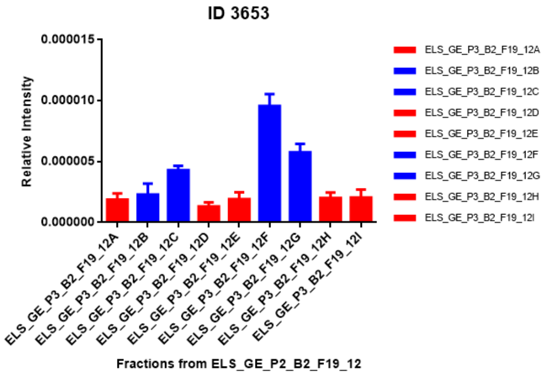	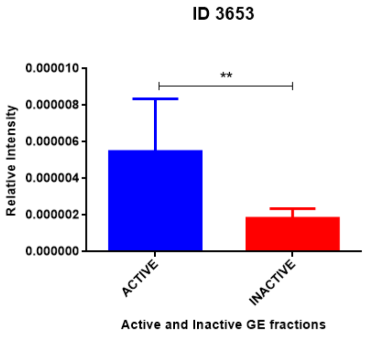

<table>
<tr><td>17871</td><td>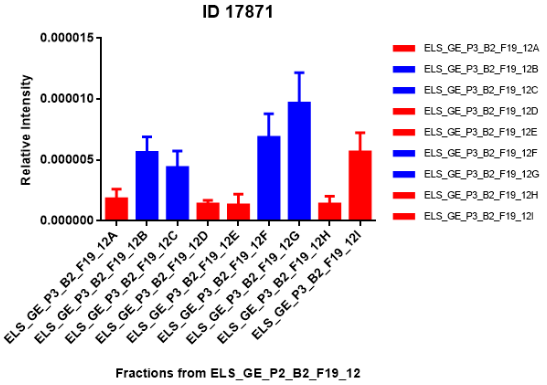
</td><td>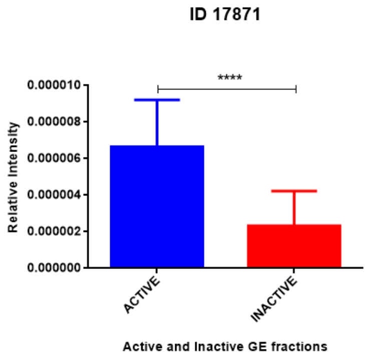
</td></tr>
<tr><td>Alignment ID</td><td>Relative Intensity Per Fraction</td><td>Relative Intensity in Active and Inactive Fractions</td></tr>
<tr><td>3618</td><td>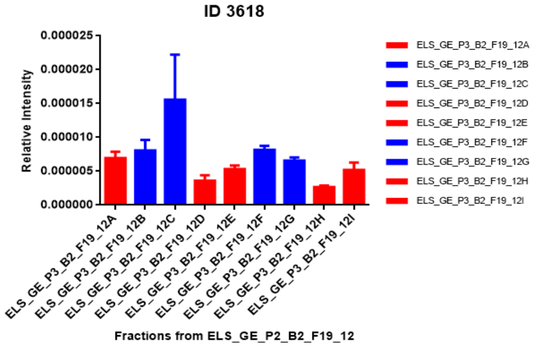
</td><td>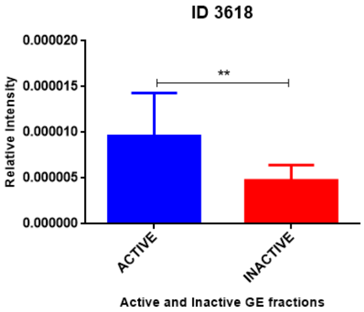
</td></tr>
<tr><td>14942</td><td>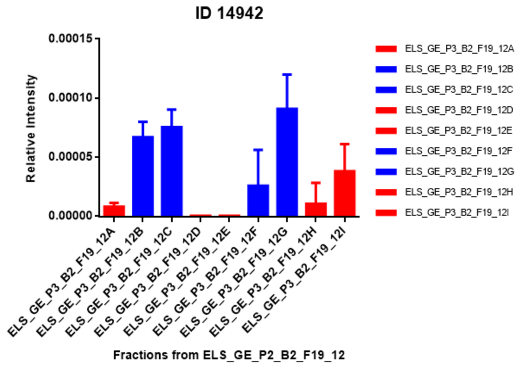
</td><td>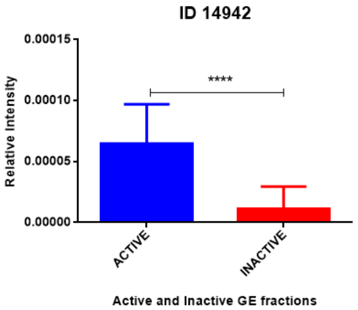
</td></tr>
</table>

3722	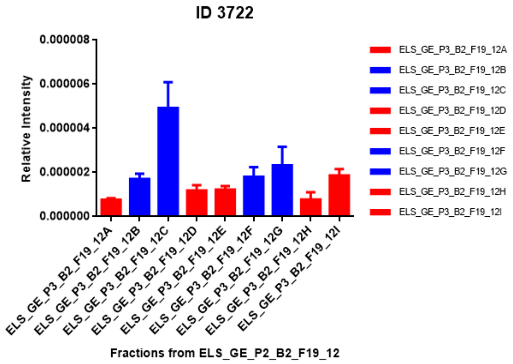	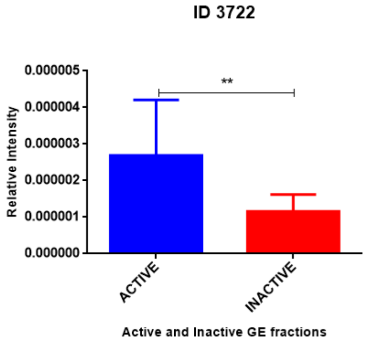
1551	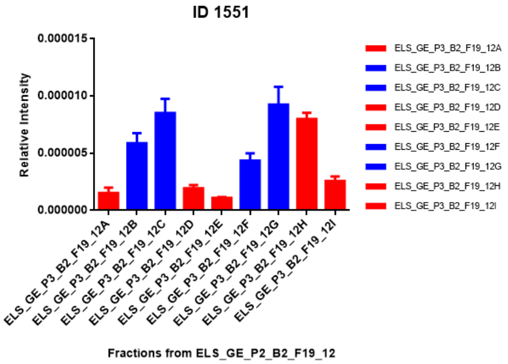	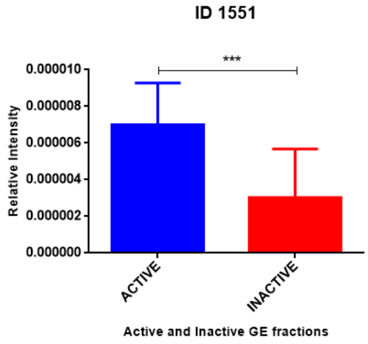
Alignment ID	Relative Intensity Per Fraction	Relative Intensity in Active and Inactive Fractions
19912	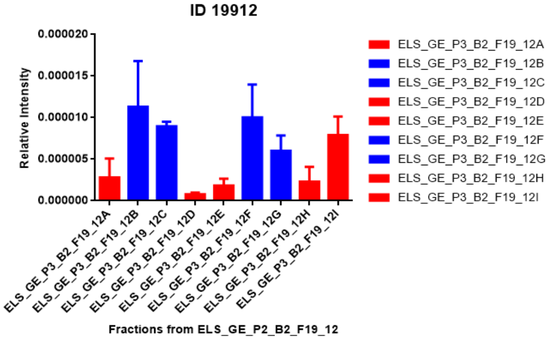	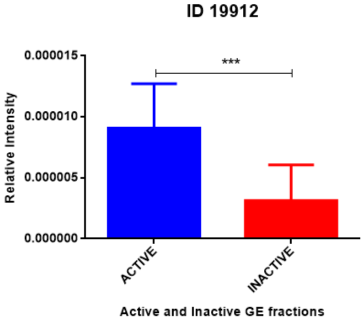
14387	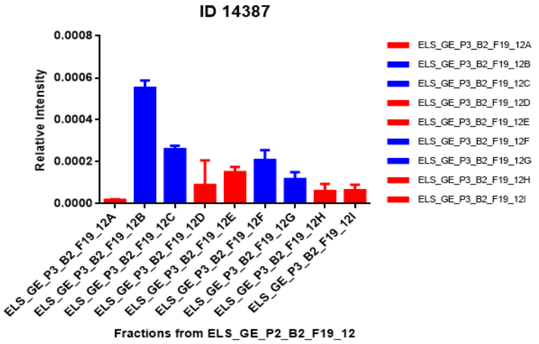	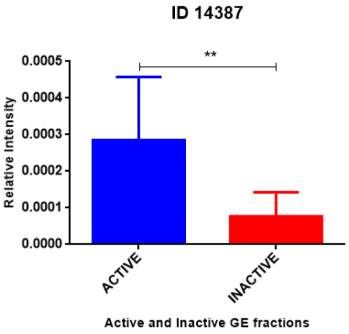

14941	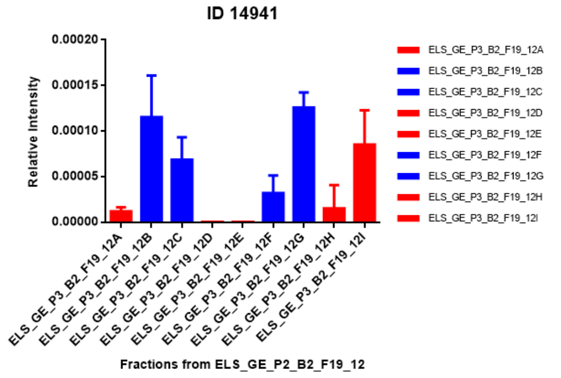	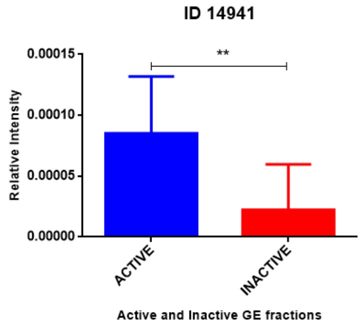
17456	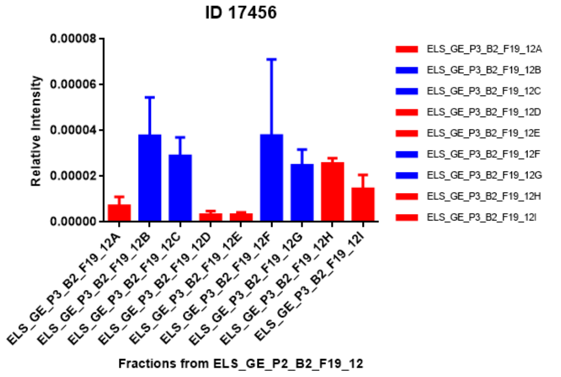	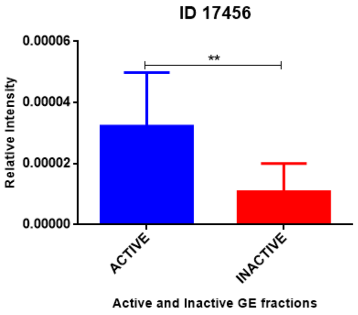

Alignment ID	Relative Intensity Per Fraction	Relative Intensity in Active and Inactive Fractions
5453	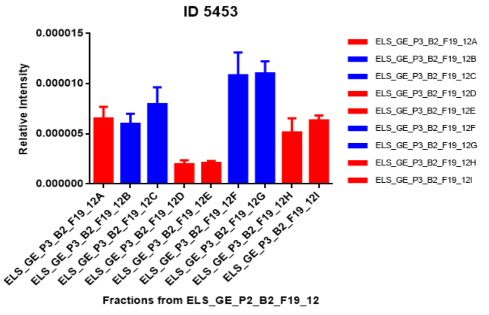	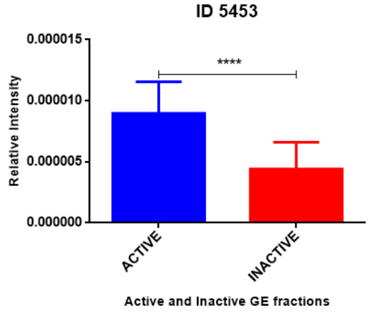
19882	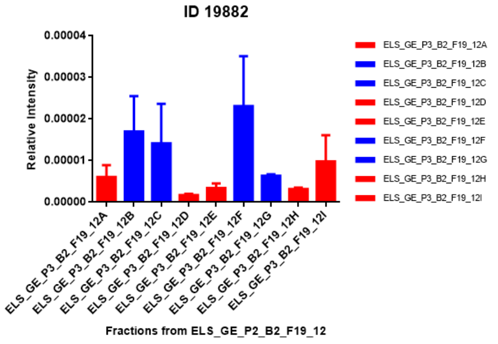	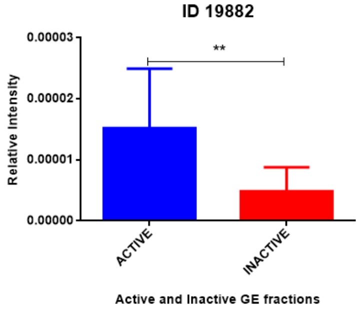

<table>
<tr><td>10807</td><td>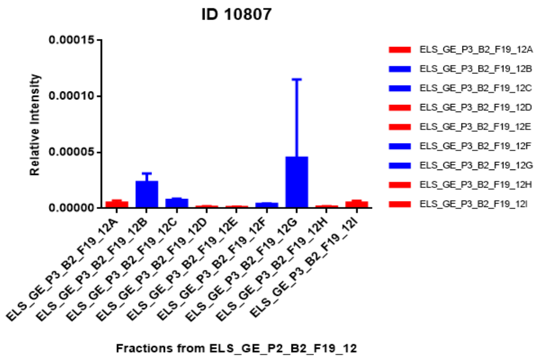
</td><td>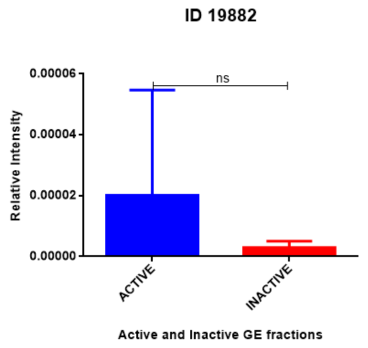
</td></tr>
<tr><td>21854</td><td>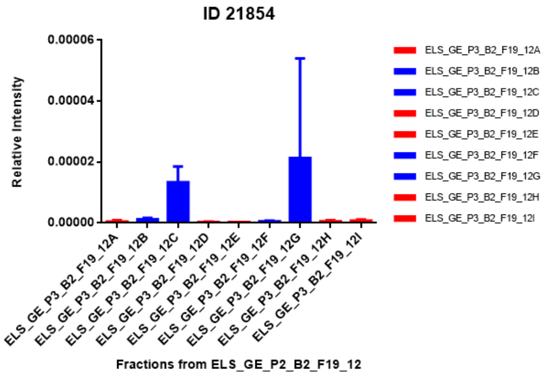
</td><td>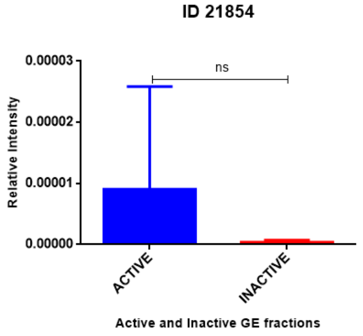
</td></tr>
<tr><td>Alignment ID</td><td>Relative Intensity Per Fraction</td><td>Relative Intensity in Active and Inactive Fractions</td></tr>
<tr><td>8693</td><td>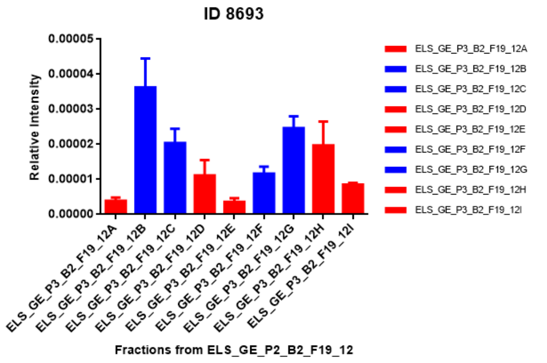
</td><td>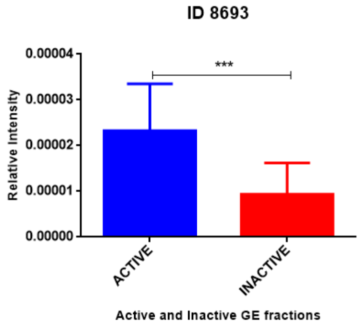
</td></tr>
</table>

6588

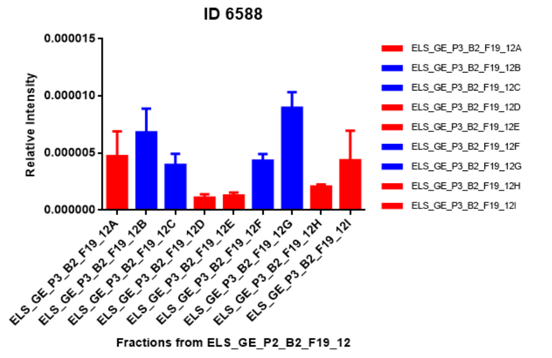

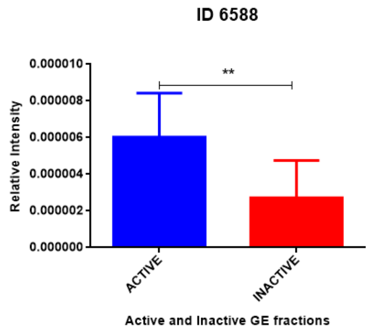

3683

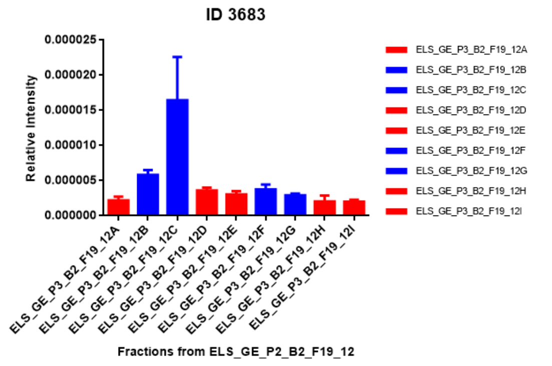

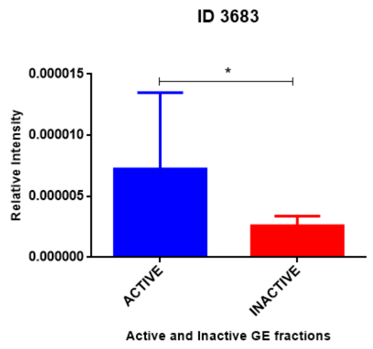

17677

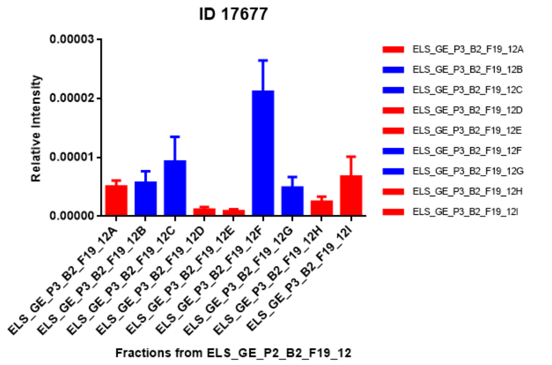

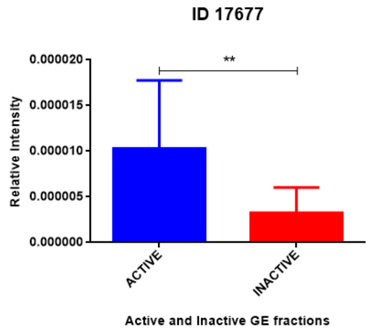

Figure S2. Putative compounds in F15-17 and their relative intensities (from MetaboAnalyst analysis).
